# What is the best technic to dislodge *Staphylococcus epidermidis* biofilm on medical implants?

**DOI:** 10.1186/s12866-022-02606-x

**Published:** 2022-08-06

**Authors:** Vivien Moris, Mylan Lam, Lucie Amoureux, Arnaud Magallon, Adrien Guilloteau, Thomas Maldiney, Narcisse Zwetyenga, Céline Falentin-Daudre, Catherine Neuwirth

**Affiliations:** 1 Department of Maxillo-Facial Surgery, Plastic, Reconstructive and Aesthetic Surgery and Hand Surgery, University Hospital of Dijon, boulevard de Maréchal-de-Lattre-de-Tassigny, 21000 Dijon, France; 2 Lipids Nutrition Cancer Team NuTox, UMR866, Université de Bourgogne Franche-Comté, 17 rue Paul Gaffarel, Dijon, 21000 France; 3 LBPS/CSPBAT, UMR CNRS 7244, Galilee Institute, Paris 13 University Sorbonne Paris Cité, 99 avenue JB, 93430 Clément, Villetaneuse France; 4 Department of Bacteriology, University Hospital of Dijon, Dijon Cedex, France; 5 UMR/CNRS 6249 Chrono-Environnement, University of Bourgogne Franche-Comté, Besançon, France; 6 Hospital Epidemiology and Hygiene Department, University of Franche-Comté, 11 Rue Claude Goudimel, Besançon, 25000 France; 7 Department of Intensive Care Medicine, William Morey General Hospital, Chalon-sur-Saône, France

**Keywords:** Biofilm quantification, Enzymatic treatment, Sonication, Medical implant, Silicone, Piccline, Peripheral venous catheter, Endotracheal tube, *Staphylococcus epidermidis*

## Abstract

**Background:**

Bacterial biofilm can occur on all medical implanted devices and lead to infection and/or dysfunction of the device.

In this study, artificial biofilm was formed on four different medical implants (silicone, piccline, peripheral venous catheter and endotracheal tube) of interest for our daily clinical and/or research practice. We investigated the best conventional technic to dislodge the biofilm on the implants and quantified the number of bacteria. *Staphylococcus epidermidis* previously isolated from a breast implant capsular contracture on a patient in the university hospital of Dijon was selected for its ability to produce biofilm on the implants. Different technics (sonication, Digest-EUR®, mechanized bead mill, combination of sonication plus Digest-EUR®) were tested and compared to detach the biofilm before quantifying viable bacteria by colony counting.

**Results:**

For all treatments, the optical and scanning electron microscope images showed substantial less biofilm biomass remaining on the silicone implant compared to non-treated implant.

This study demonstrated that the US procedure was statistically superior to the other physical treatment: beads, Digest-EUR® alone and Digest-EUR® + US (*p* < 0.001) for the flexible materials (picc-line, PIV, and silicone). The number of bacteria released by the US is significantly higher with a difference of 1 log on each material. The result for a rigid endotracheal tube were different with superiority for the chemical treatment dithiothreitol: Digest-EUR®. Surprisingly the combination of the US plus Digest-EUR® treatment was consistently inferior for the four materials.

**Conclusions:**

Depending on the materials used, the biofilm dislodging technique must be adapted. The US procedure was the best technic to dislodge *S. epidermidis* biofilm on silicone, piccline, peripheral venous catheter but not endotracheal tube. This suggested that scientists should compare themselves different methods before designing a protocol of biofilm study on a given material.

**Supplementary Information:**

The online version contains supplementary material available at 10.1186/s12866-022-02606-x.

## Introduction

The biofilm is the most common form found in nature for many bacterial species. To increase their probability of survival in their environment, bacteria secrete a layer of extracellular polymeric substances (EPS) [1–3]. The particular architecture of the biofilm effectively protects the bacteria from external environmental aggressions such as UV irradiation, antibiotics and disinfection. These bacterial species are more tolerant than planktonic bacteria [4–6]. Biofilm communities can harbor tolerant and persister cells, these that can survive transient antibiotic treatment and that regrow when the antibiotic is withdrawn [7]. These characteristics make it difficult to remove the biofilm. Several methods have been reported for the analysis of biofilms [3]. If biofilm persists on surgical instruments or medical implants, living bacteria can lead to hospital-acquired infections, resulting in public health problems and increased hospital costs [8, 9]. For example, flexible endoscopes used in gastroenterology are ideal surfaces for biofilm growth. Many viable bacteria have been found on endoscopes despite the cleaning, disinfection and sterilization process in hospitals. [10, 11].

From clinical point of view, biofilm occurs in several situation. For instance biofilm growth occurs in the lungs of cystic fibrosis patients [12]. The biofilm structure acts as a shield and protects the bacteria from the antimicrobials. In patients undergoing mechanical ventilation, the formation of biofilm on endotracheal tubes is an early and frequent event. Moreover, high-grade biofilm formation on an endotracheal tube is associated with the development of ventilator-associated pneumonia [13].

Regarding infections associated with biomaterials (BAI), the main source of contamination is the patient's skin. The bacterial flora of human skin consists mainly of *Staphylococcus epidermidis* and *Staphylococcus aureus.* When a medical device is implanted, contact with the skin is sufficient to contaminate the implant [14]. Fragile patients with comorbidities are the most susceptible to nosocomial infections. All implants are at risk of being colonized by bacteria. Studies find 60%-70% of nosocomial infections caused by contaminated medical implants [15]. Contamination of the medical implant can lead to device malfunction, systemic infection by hematogenous spread of the bacterial agent, and even to tissue destruction resulting in severe disease and death [16].

All medical implants are at risk of bacterial colonization and infection such as cardiac prostheses, orthopedic implants, silicone breast implants, dental implants, intravascular catheters, artificial pumps left ventricular assist devices, pacemakers, vascular prostheses, cerebrospinal fluid shunts, urinary catheters, voice prostheses, ocular prostheses, contact lenses and intrauterine contraceptive devices [16, 17].

Several challenges are encountered when attempting to treat infections related to biofilms covering medical implants. These include chronic infection, impaired wound healing and acquired antibiotic resistance. The biofilm grows and can lead to the dissemination of infectious emboli [15–17]. When an implant is placed, the human body identifies the implant as a foreign body. A physiological balance is established between the host (the human body) and the implant. This phenomenon, called biocompatibility, can be seriously compromised if bacteria adhere to the surface of the implant, which can lead to a form of rejection of the implant [18]. For example, infections related to orthopedic implants can result in osteomyelitis with destruction of the bone and surrounding soft tissue. Bone is a very poorly vascularized tissue, which makes treatment of these infections with antibiotics difficult and ineffective [19–22]. Thus, treatment of infections in orthopedic devices requires a multi-step procedure. In the first stage, the infected implant is removed, the patient is treated for infection, and then a new device is implanted in the second stage when no further signs of infection are present. This multi-stage procedure results in high morbidity with bed rest, cardiovascular problems and difficulty walking.

Capsular contracture (CC) is the contraction of fibrotic scar tissue around the silicone breast implant. It is the most common complication of breast augmentation. It can lead to asymmetry, pain, and its treatment requires a surgical revision [23]. Studies have reported incidence rates of CC ranging from 5 and 19% [24, 24, 25]. The fibrotic tissue around the implants was analyzed by scanning electron microscopy confirming the presence of bacterial biofilm. The most common germ found in capsular contracture was *Staphylococcus epidermidis* [26]. The severity of capsular contracture is assessed according to the Baker scale. It has been shown that the higher the Baker grade, the higher the number of bacteria in the human periprosthetic capsule [27] and in the porcine model [28]. In 2011, the FDA alerted to a strong association between large cell anaplastic lymphoma (BIA-ALCL) and textured breast implants [29]. This is a rare non-Hodgkin's T-cell or null lymphoma first described by Stein and colleagues [30]. The clinical symptomatology of this pathology is common and misleading with the appearance of a late peri-implant seroma (the pathology occurs on average after 8 years of implant placement) containing malignant cells in one breast. Occasionally, a tumor mass attached to the capsule may be found. Lymph node involvement is found in 5 to 10% of patients. The pathophysiology is not yet elucidated, but a serious hypothesis focuses on infection by the biofilm, associated with a genetic predisposition of the patient. The chronic inflammation caused by the periprosthetic bacterial biofilm activates the immune response, which activates T lymphocytes and triggers polyclonal proliferation. This chronic inflammation can lead to monoclonal proliferation of T lymphocytes, which can lead to the development of ALCL [31]. It was found that bacterial adhesion to silicone is significantly higher than to polyurethane or Teflon [32].

To avoid as much as possible this kind of complications, new materials limiting the adhesion of bacteria are currently being studied. The prevention of biofilm formation in medical implants can be controlled by following various novel emergent strategies like polymer coatings, antimicrobial coatings, nanostructured coatings, surface modifications, and biosurfactants. Non antibiotic based therapies are proposed such as enzyme-mediated approaches, phage therapy or immunotherapy, [33].

Nevertheless, it is essential to correctly quantify the biofilm on new biomaterials to determine their ability to avoid biofilm formation and to compare them with the current ones.

### Biofilm analysis

Several approaches have been developed to study biofilm [34], including bacterial counting, colorimetric methods with dyes (crystal violet, SYTO9 staining) and imaging methods such as optical microscopy, electron microscopy, fluorescence microscopy, and confocal microscopy. These methods provide different kind of information that seem sometimes incoherent. For instance a discrepancy between biofilm size and number of viable bacteria has been reported [35]. Moreover, biofilms are spatially heterogeneous, and microscopy inherently becomes biased by the regions selected for imaging as it would be cost and time prohibitive to image an entire surface with the resolution necessary for cell counting. On the other hand, colony enumeration in theory represents a count of the entire substrate.

The adhesion of microorganisms to prosthetic surfaces reduces their detection [36]. Therefore, to measure viable bacteria present in the biofilm, detaching efficiently the biofilm surrounding the implants is essential. The detachment procedure must effectively detach and separate individual cells to generate reliable colony forming units (CFU) values [37] while maintaining their cultivability [3, 34]. Furthermore, most studies use scrapping, enzymatic or ultrasonic detachment procedures [38–40]. Despite microbiology culture techniques’ play a key role in diagnosing these complex implant-related infections there is a universal lack of standardized and shared procedures for microbiological sampling and processing [36].

### Biofilm removal methods

- Sonication is ultrasonic energy applied to the biomaterial surface to disrupt adherent biofilm [38]. There are two types of sonication: direct sonication via a tip coming into direct contact with the implant and indirect sonication with the implant placed in a water bath. In this study, the indirect sonication method was used.
- Enzymatic techniques attempt to break chemical bonds in the extracellular matrix of the biofilm to detach bacteria [40]. Digest-EUR® is a mucolytic composed of dithiothreitol for rapid digestion and mucus fluidification [41].
- Mechanized bead mill process: the implants are placed in a sterile tube with 3 ml of distilled water and 1 mm-diameter stainless steel beads before the agitation bead mill (6000 rpm) with the Ultra Turrax® Tube Drive disposal [42].

In this study, artificial biofilm was formed on four different medical implants of interest for our daily clinical and/or research practice. We investigated the best conventional technic to dislodge the biofilm on the implants and quantify the number of bacteria. The type of implants selected for the study included i.silicone implants, ii catheters and iii.endotracheal tube.

- *i*. Silicone implants are widely used for breast augmentation and breast reconstruction. Bacterial biofilms have been implicated with breast implant complications, including capsular contracture [24, 25], and breast implant-associated anaplastic large-cell lymphoma (BI-ALCL) [43].
- *ii*. Catheter related infections are a major cause of morbidity and mortality worldwide. In the United States, 250,000 hospital-acquired bloodstream infections per year have been reported and 23,000 related to central venous catheter infection in 2009 [44]. Another study conducted in the USA reported a mortality rate of 27% in catheter-associated bacteremia (all types) [45]. A peripherally inserted central venous catheter (PICC-line) is an intravenous access that can be used for a prolonged period for chemotherapy regimens, extended antibiotic therapy, or total parenteral nutrition.
- *iii.* Endotracheal tubes are used daily for ventilation during surgery under general anesthesia but also in intensive care units for invasive ventilation. High-grade biofilm formation on an endotracheal is associated with the development of ventilator-associated pneumonia [13, 46].

## Results

### Microscopy

**Fig. 1:**
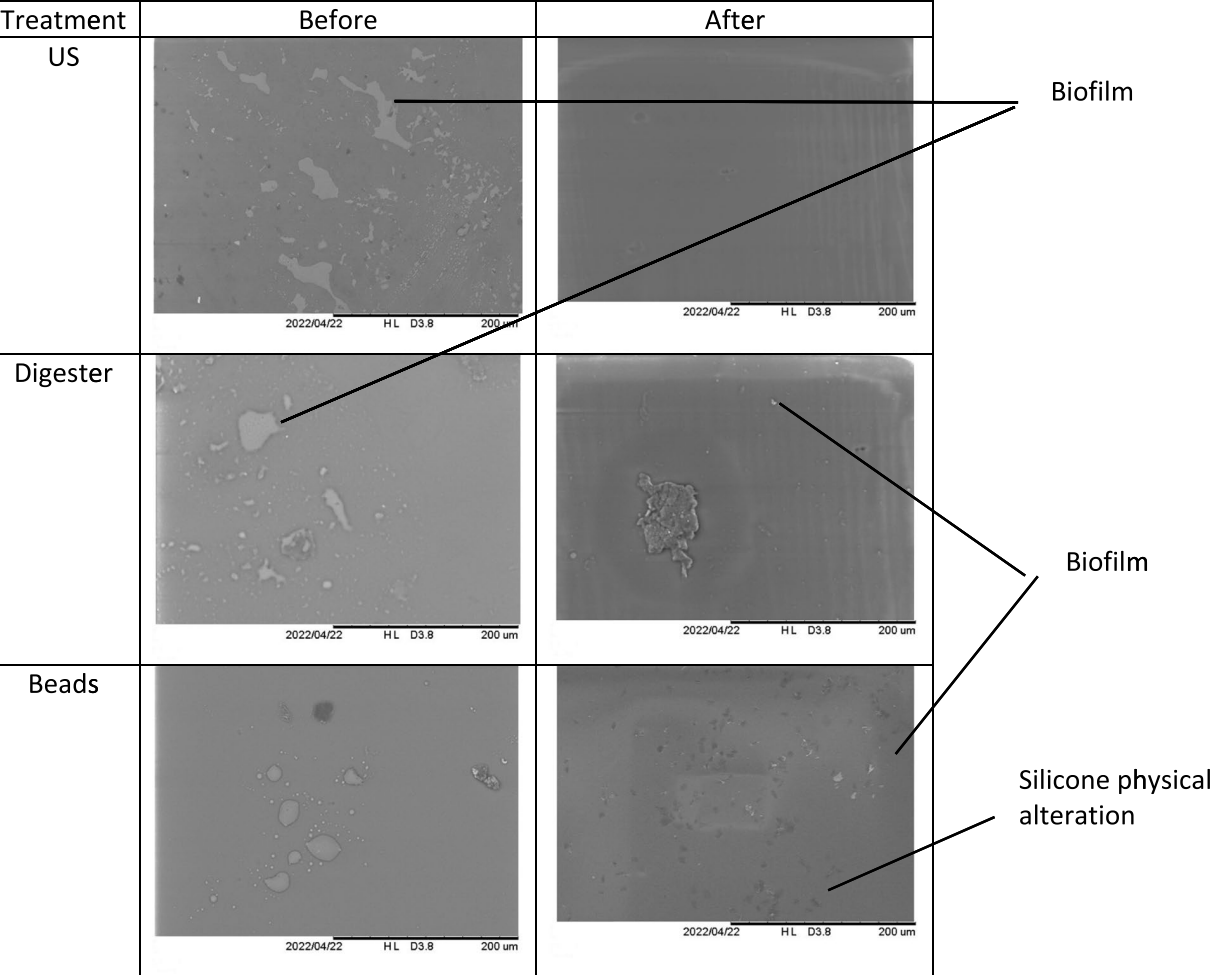
Scanning electron microscopy (200 µm) shows the formation and removal of biofilms (white spots on images) on silicone for Staphylococcus epidermidis

**Fig. 2:**
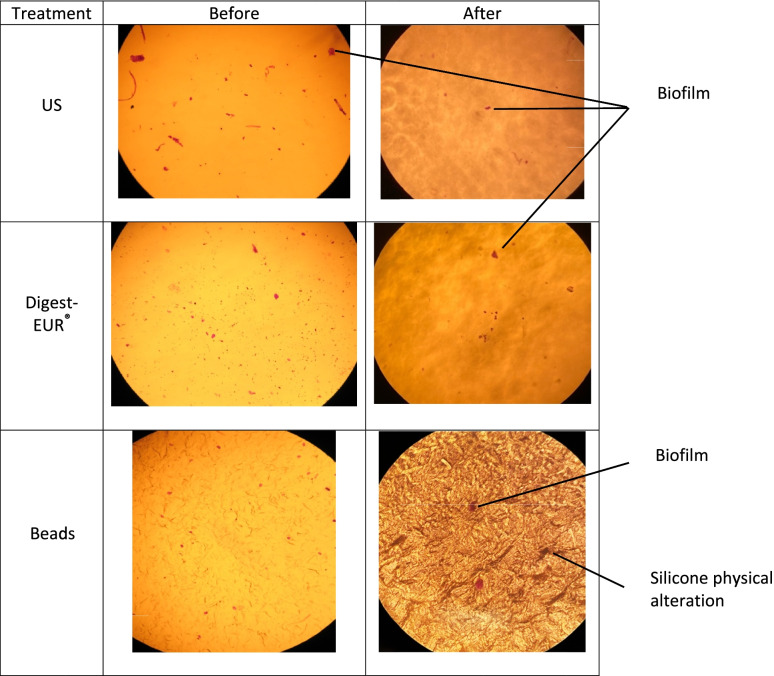
Optical microscopy (X10) shows the formation and removal of biofilms (pink color on images) on silicone for Staphylococcus epidermidis

The use of optical microscopy (OM) and scanning electron microscopy (SEM) allowed to visualize the biofilms of *S. epidermidis* before and after treatments with either chemicals, sonication or bead mill processing. For all treatments, the optical and scanning electron microscope images showed substantial less biofilm biomass remaining on the silicone implant compared to non-treated implant (Fig. 1). All materials showed physical alteration on the OM and SEM images after beads treatment as shown on Fig. 1 and 2 for silicone.

**Fig. 3:**
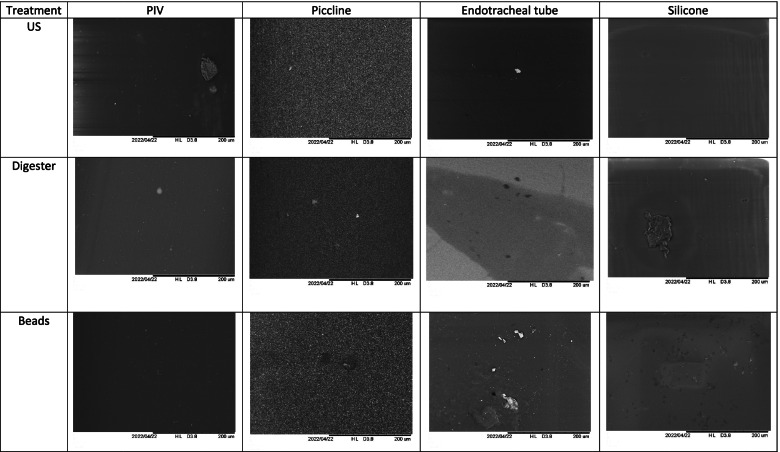
Scanning electron microscopy (200 µm) shows the residual biofilms (white spots on images) on different biomaterials for Staphylococcus epidermidis after each treatment

The Fig. 3 showed the residual *Staphylococcus epidermidis* biofilms on different biomaterials after each treatment.

### Numeration

The effect of the different procedures on the biofilms removal was evaluated by means of the bacterial CFUs in the culture supernatants. Two duration of biofilm were performed, 14 h and 5 days.

**Fig. 4:**
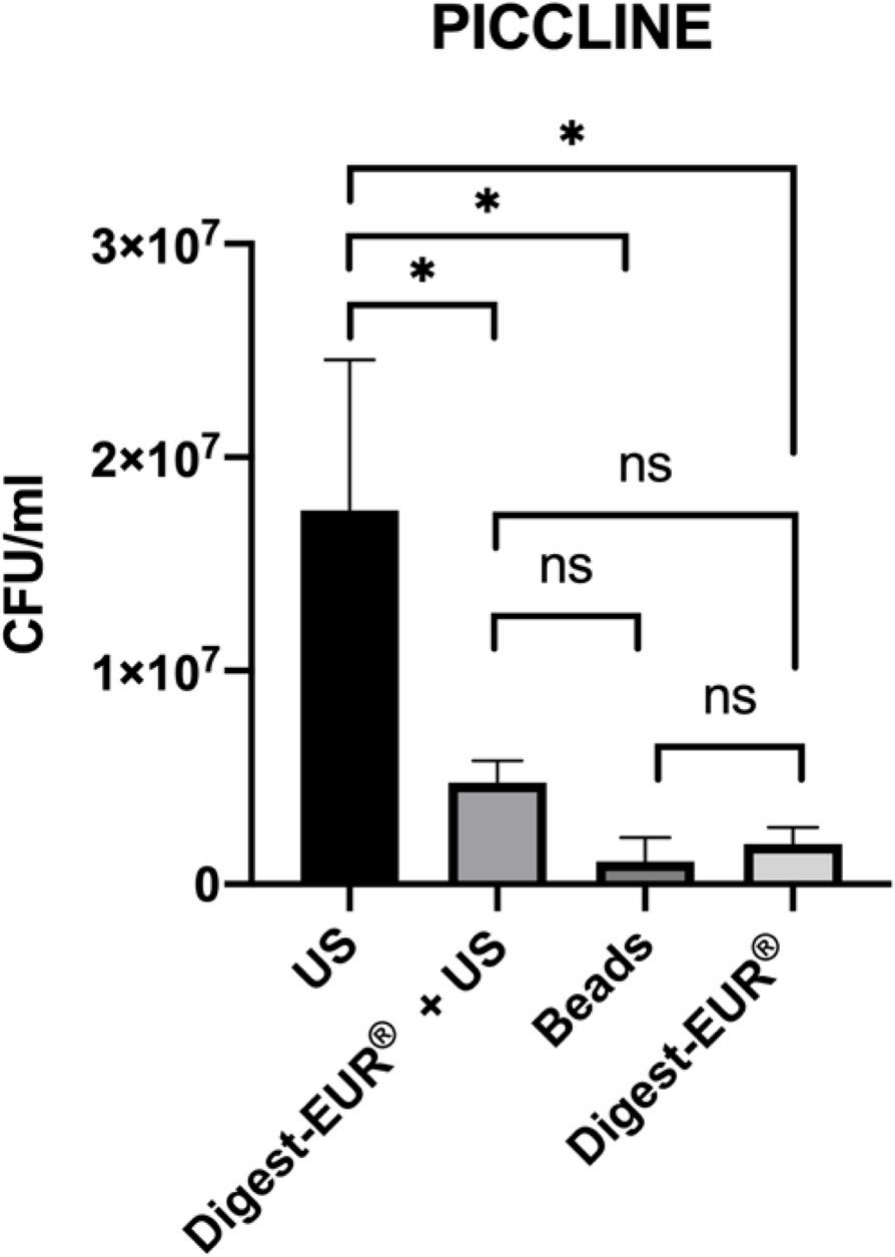
Comparison of biofilm removal procedure on piccline. US procedure dislodged 1.75 × 107 CFU/ml, Digest-EUR® and US dislodged 4.75 × 106 CFU/ml, beads dislodged 1.06 × 106 CFU/ml and Digest-EUR® dislodged 1.9 × 106 CFU/ml. *: *p* < 0.001 / ns: *p* > 0.05

**Fig. 5:**
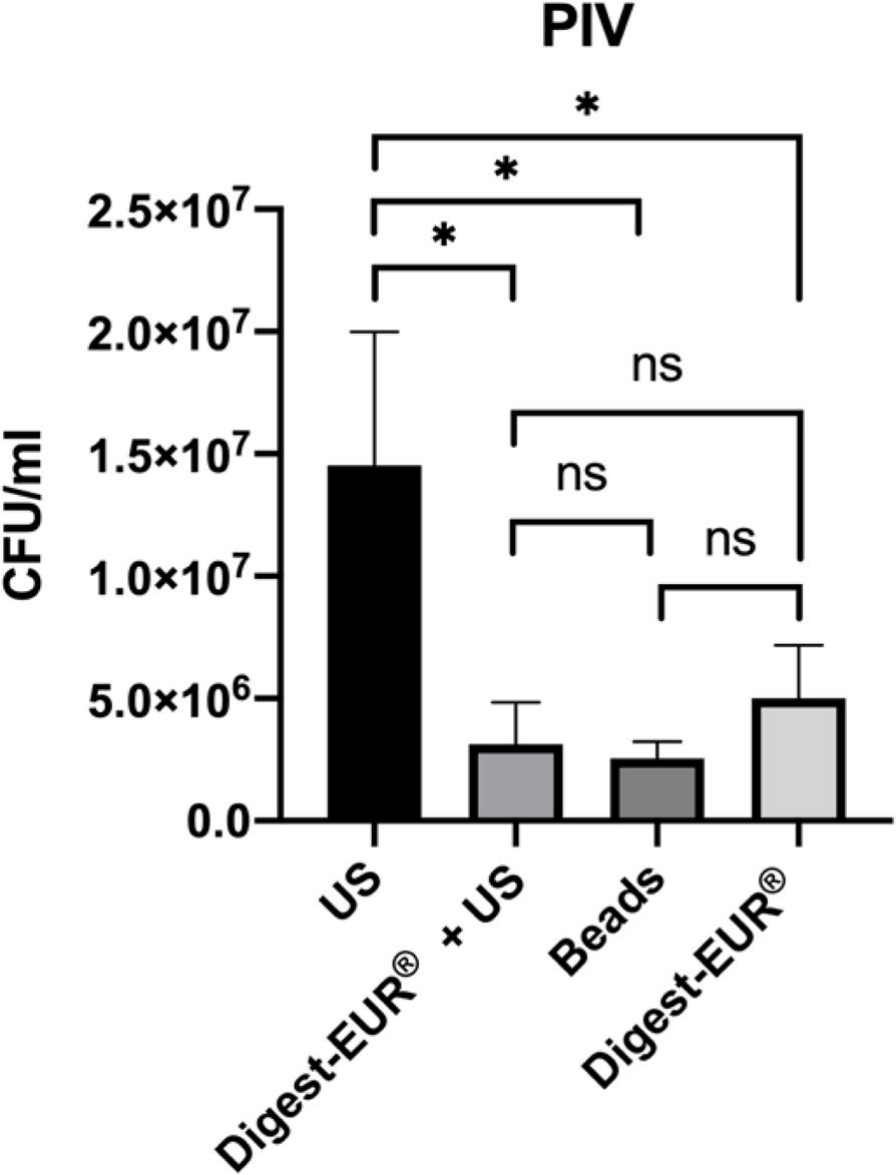
Comparison of biofilm removal procedure on Peripheral Intravenous Catheter (PIV). US procedure dislodged 1.45 × 107 CFU/ml, Digest-EUR® and US dislodged 3.13 × 106 CFU/ml, Beads dislodged 2.55 × 106 CFU/ml and Digest-EUR® dislodged 5.01 × 106 CFU/ml. *: *p* < 0.001 / ns: *p* > 0.05

**Fig. 6:**
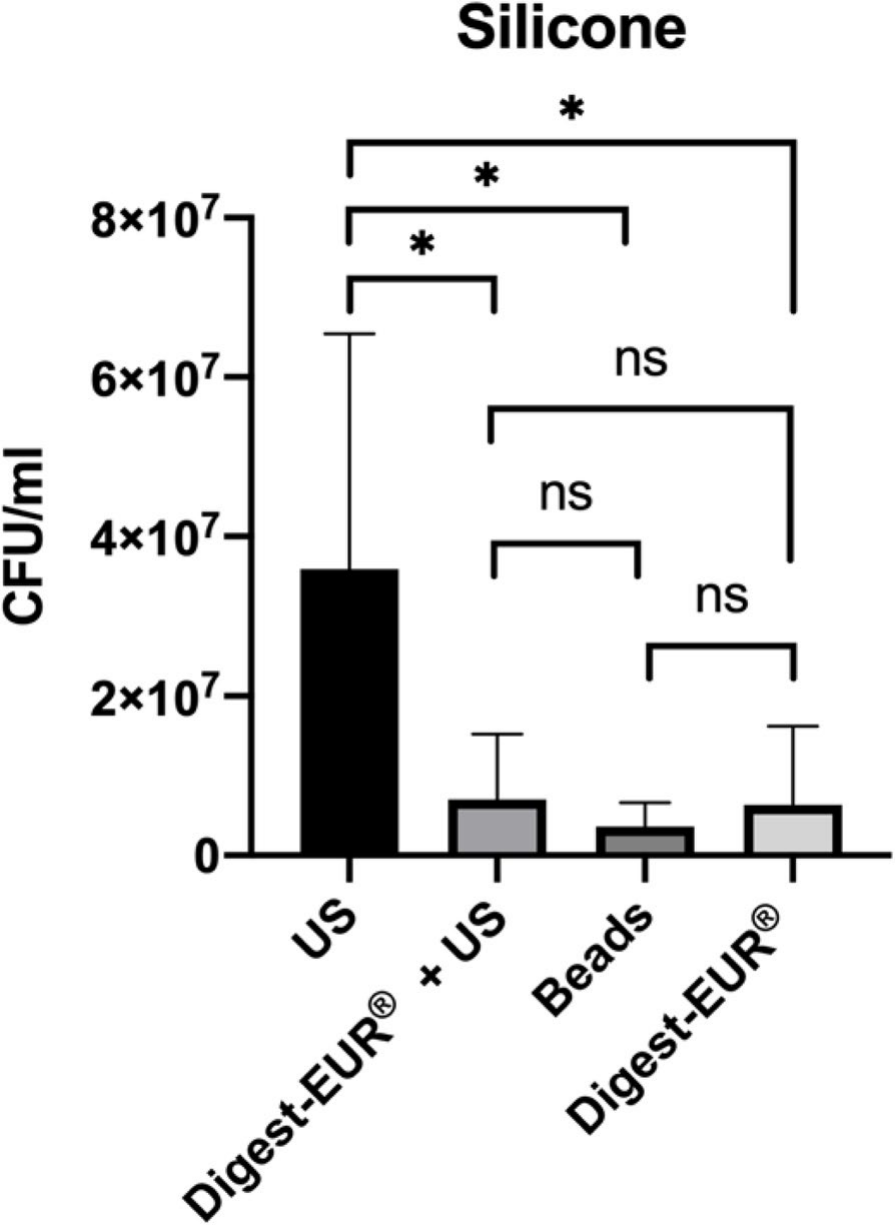
Comparison of biofilm removal procedure on silicone. The US procedure dislodged 3.59 × 10^7^ CFU/ml, Digest-EUR® and US dislodged 7 × 10^6^ CFU/ml, Beads dislodged 3.61 × 10^6^ CFU/ml and Digest-EUR® dislodged 6.36 × 10^6^ CFU/ml. *: *p* < 0.001 / ns: *p* > 0.05

With the 14 h *Staphylococcus *epidermidis biofilm results shows:

- For the picc-line, the US procedure dislodged 1.75 × 10^7^ CFU/ml (SD: 7.07 × 10^6^ CFU/ml); the second-best technic was the combination of Digest-EUR® and US with 4.75 × 10^6^ CFU/ml, which is 73% less effective than the US technic alone (Fig. 4).
- For the PIV, the US procedure dislodged 1.45 × 10^7^ CFU/ml (SD: 5.46 × 10^6^ CFU/ml); the second-best technic was the Digest-EUR® with 5.01 × 10^6^ CFU/ml, which is 66% less effective than the US technic alone (Fig. 5).
- For the silicone, the US procedure dislodged 3.59 × 10^7^ CFU/ml (SD: 2.95 × 10^7^ CFU/ml); the second-best technic was the combination of Digest-EUR® and US with 7 × 10^6^ CFU/ml, which is 80% less effective than the US technic alone (Fig. 6).

The US procedure was statistically superior to the other physical treatment: Digest-EUR® + US, beads, and Digest-EUR® alone (*p* < 0.001) for the picc-line, PIV, and silicone.

**Fig. 7:**
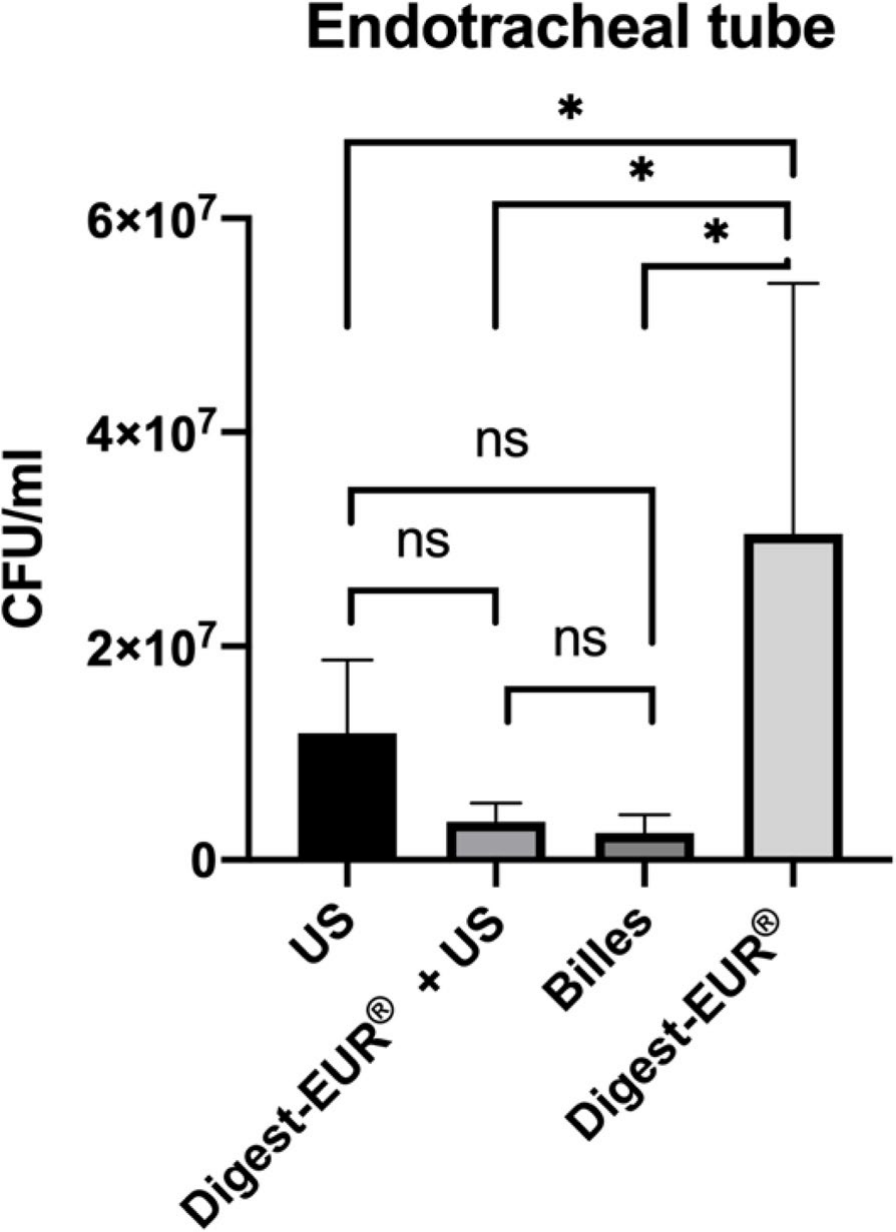
Comparison of biofilm removal procedure on endotracheal tube. the US procedure dislodged 1.19 × 107 CFU/ml, Digest-EUR® and US dislodged 3.59 × 106 CFU/ml, Beads dislodged 2.53 × 106 CFU/ml and Digest-EUR® dislodged 3.05 × 107 CFU/ml. *: *p* < 0.001 / ns: *p* > 0.05

The result for the endotracheal tube was different with superiority for the Digest-EUR® treatment with 3.05 × 10^7^ CFU/ml (SD: 2.35 × 10^7^ CFU/ml) (*p* < 0.05), US treatment was the second-best technique to dislodged biofilm on the endotracheal tube (Fig. 7).

**Fig. 8:**
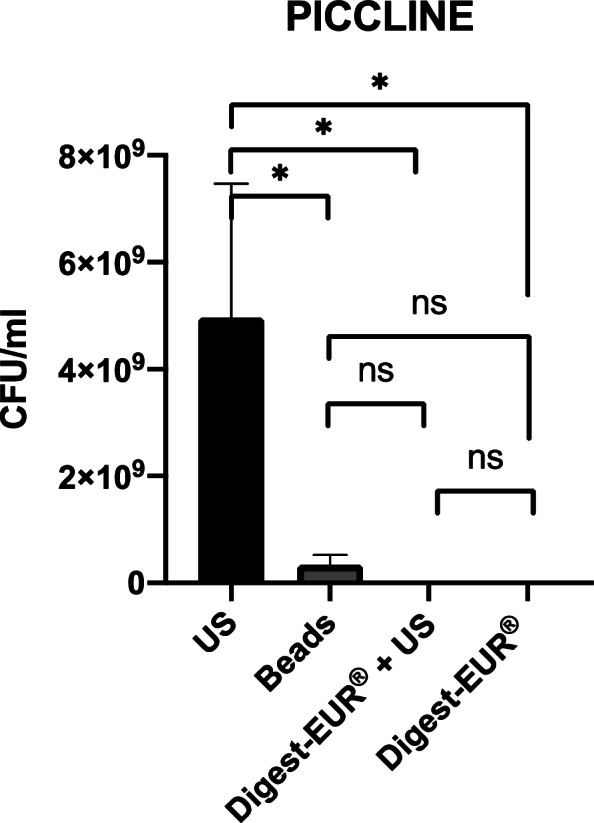
Comparison of a 5 days biofilm removal procedure on piccline. US procedure dislodged 5 × 10^9^ CFU/ml, Digest-EUR® and US dislodged 1.2 × 10^7^ CFU/ml, beads dislodged 3.4 × 10^8^ CFU/ml and Digest-EUR® dislodged 3.03 × 10^6^ CFU/ml. *: *p* < 0.001 / ns: *p* > 0.05

**Fig. 9:**
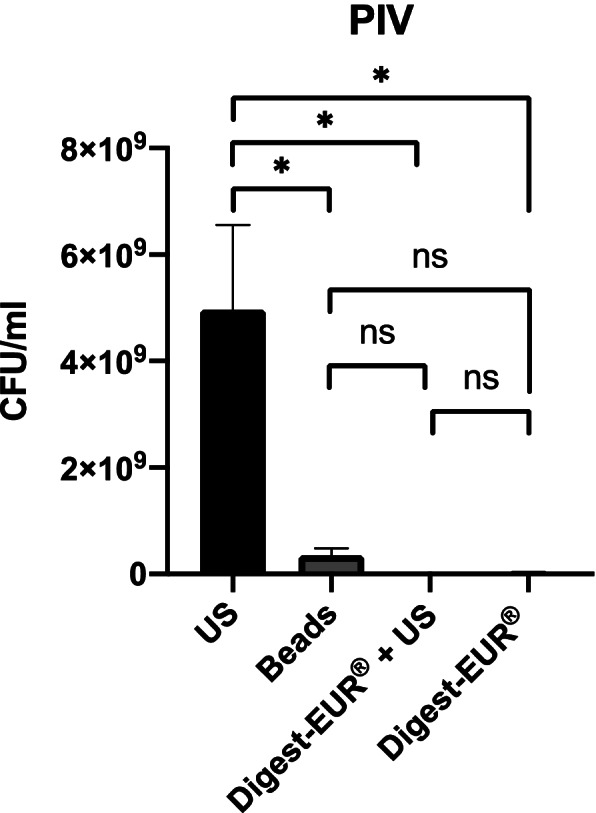
Comparison of a 5 days biofilm removal procedure on Peripheral Intravenous Catheter (PIV). US procedure dislodged 5 × 10^9^ CFU/ml, Digest-EUR® and US dislodged 1.15 × 10^7^ CFU/ml, Beads dislodged 3.53 × 10^8^ CFU/ml and Digest-EUR® dislodged 3.02 × 10^7^ CFU/ml. *: *p* < 0.001 / ns: *p* > 0.05

**Fig. 10:**
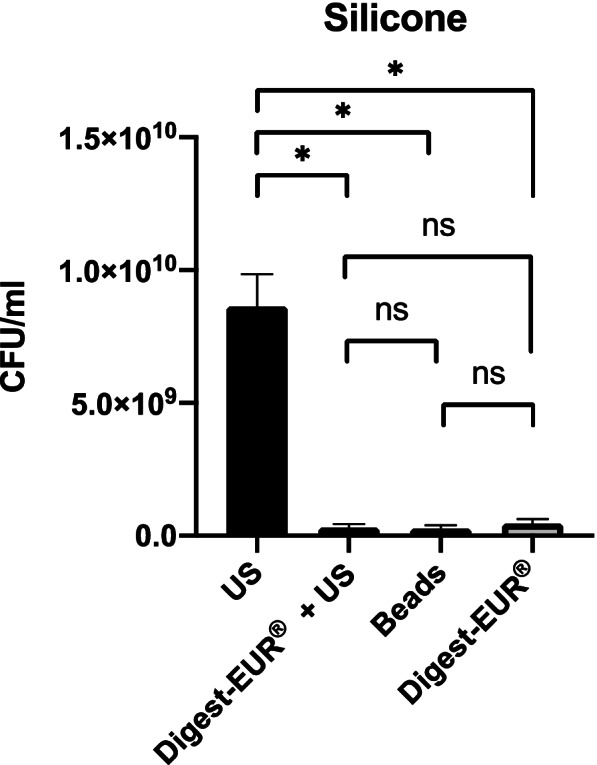
Comparison of a 5 days biofilm removal procedure on silicone. The US procedure dislodged 8.63 × 10^9^ CFU/ml, Digest-EUR® and US dislodged 3.01 × 10^8^ CFU/ml, Beads dislodged 2.74 × 10^8^ CFU/ml and Digest-EUR® dislodged 4.52 × 10^8^ CFU/ml. *: *p* < 0.001 / ns: *p* > 0.05

With the 5 days *Staphylococcus epidermidis* biofilm results shows:

- For the picc-line, the US procedure dislodged 5 × 10^9^ CFU/ml (SD: 2.5 × 10^9^ CFU/ml) (Fig. 8).
- For the PIV, the US procedure dislodged 5 × 10^9^ CFU/ml (SD: 1.6 × 10^9^ CFU/ml) (Fig. 9).
- For the silicone, the US procedure dislodged 8.63 × 10^9^ CFU/ml (SD: 1.22 × 10^9^ CFU/ml) (Fig. 10).

The US procedure was statistically superior to the other physical treatment: Digest-EUR® + US, beads, and Digest-EUR® alone (*p* < 0.001) for the picc-line, PIV, and silicone.

**Fig. 11:**
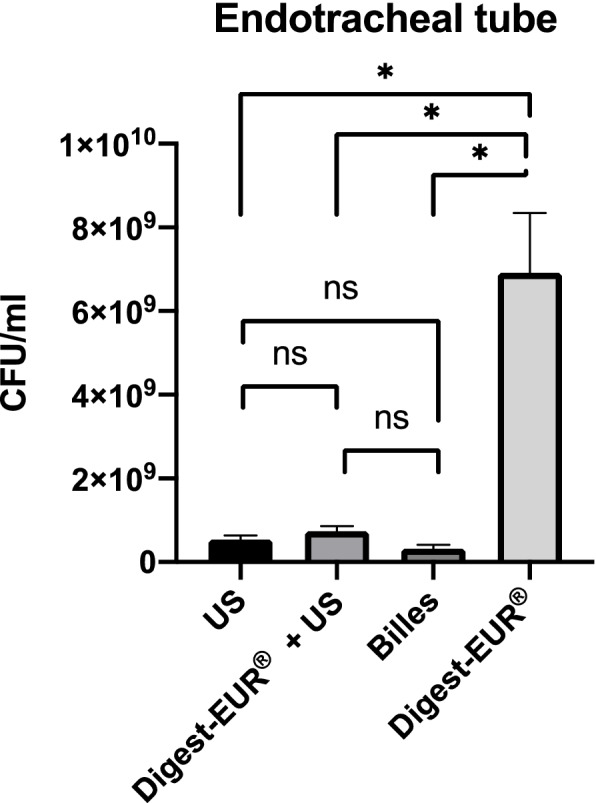
Comparison of a 5 days biofilm removal procedure on endotracheal tube. the US procedure dislodged 5.34 × 10^8^ CFU/ml, Digest-EUR® and US dislodged 7.41 × 10^8^ CFU/ml, Beads dislodged 3.18 × 10^8^ CFU/ml and Digest-EUR® dislodged 6.91 × 10^9 CFU^/ml. *: *p* < 0.001 / ns: *p* > 0.05

The result for the endotracheal tube was different with superiority for the Digest-EUR® treatment with 6.91 × 10^9^ CFU/ml (SD: 1.43 × 10^9^ CFU/ml) (p < 0.05) (Fig. 11).

The flow cell cytometry (Sysmex®) was used to confirm a correct single cell dispersal. Samples were vortexed and then passed through the cytometer 5 times. Counts varied by a maximum of 1.5 times, showing little heterogeneity.

## Discussion

This study assessed the best physical technic to detach the biofilm on the implants and quantify the number of bacteria via a direct counting method. There are still limited data on biofilm removal capacity using different protocols, devices, and chemicals [47].

Indeed, a new polymer coating on silicone is currently in development and we are in charge of studies dealing with its biocompatibility and its ability to reduce biofilm formation. Prior to these experiments it was necessary to evaluate which method is the most efficient to dislodge biofilm.

This work focused specifically on Staphylococcus epidermidis biofilm.

Indeed S. epidermidis, which is an important component of the cutaneous flora, is a very common causative agent of acute and chronic prosthetic infections with long-term effects [9]. The clinical strain we employed had a priori demonstrated its ability to establish clinically relevant biofilm infections on breast implants. Several options were available to evaluate the amount of biofilm developed on medical implants. The strategy of bacterial counting was retained for its clinical relevance with two arguments. First this is the reference method for most bacteriological analyses performed in all laboratories in charge of clinical specimen. It allows counting live cells capable of forming a colony, performing isolates identification and antibiotic susceptibility testing. Second it is performant to detect several bacterial species within the same sample. Imaging technics were used to ensure that biofilm developed and to visualize the effect of the different detachment treatments. Using only imaging provides information about the depth of the structure and in case of use of vital dyes an estimation of the number of viable bacteria. Nevertheless, the level of accuracy of imaging is much lower than conventional bacterial counting [48].

However, it should be noted that the CFU does not allow the quantification of extracellular polymeric substances or dead bacteria. EPS and dead bacteria also play a role in the difficulty of treating infections with biofilm. They are obstacles for a good penetration of antibiotics in living bacteria [49].

The use of crystal violet has proven extremely useful as a cell estimate for biofilm growth [50, 51]. However we chose not to investigate biofilm formation by means of dyes because we observed that the silicone captured the crystal violet by himself as already noticed [52].

Interestingly images revealed that beads treatment is aggressive: the silicone shows physical alteration on the OM and SEM images. The use of beads in bacteriology is routinely used to prepare infected bone samples for the detection of germs [53]. This technique has shown disappointing results for the biomaterials used in our study. It is possible that the shocks caused by the beads have the opposite effect of sticking the biofilm stronger on the biomaterials.

The US procedure was statistically superior to the other physical treatment: Digest-EUR® + US, beads, and Digest-EUR® alone (*p* < 0.001) for the picc-line, PIV, and silicone. The number of bacteria released by the US is significantly higher with a difference of 1 log on each material. The first results with a 14 h biofilm are confirmed and accentuated with the 5 day biofilm.

Previous studies have found that sonication and vortexing increase the number of bacteria isolated from joint implants [54–56]. In patients undergoing knee or hip revision surgery, Trampuz et al. found that a culture of samples obtained after sonication was 18% more sensitive than the traditional culture of periprosthetic tissue. This sensitivity was even higher in patients who had received antibiotics in the 14 days prior to surgery 30% more sensitive [54].

The US procedure is simple, quick, and effective. In the present study the sonication was performed during one minute according to previous results. Indeed Kobayashi et al. (2007) recommended a sonication time of between 1 and 5 min as being ideal for dislodging biofilm bacteria without affecting bacterial viability [57]. The duration of the sonication time has already been evaluated on PMMA (Poly-méthyl-méthacrylate), one minute was already sufficient to dislodge all bacteria [57]. It has been reported that long durations of sonication damage bacterial viability [58].

The result for the endotracheal tube was different with superiority for the Digest-EUR® treatment with 3.05 × 107 CFU/ml (SD: 2.35 × 107 CFU/ml) (*p* < 0.05), US treatment was the second-best technique to dislodge biofilm on the endotracheal tube (Fig. 7). Different chemical treatments N-acetyl cysteine (NAC) and dithiothreitol (DTT) (Digest-EUR®), have been evaluated by other authors. They found that treatment with Digest-EUR® provided a greater bacterial recovery rate than those obtained with NAC treatment and scraping, similar to that observed with sonication [59]. In the present study sonication proved to be less efficient than Digest-EUR®.

We hypothesized that better results might be obtained by combinations of treatments.

However, the results show that the combination of the US plus Digest-EUR® treatment was consistently inferior. For example, for the silicone, the US procedure dislodged 3.59 × 107 CFU/ml (SD: 2.95 × 107 CFU/ml); the Digest-EUR® dislodged 6.36 × 106 CFU/ml (SD: 9.87 × 106 CFU/ml) and the combination of Digest-EUR® and US dislodged 7 × 106 CFU/ml (SD: 8.25 × 106 CFU/ml), which is 80% less efficient than the US technic alone. The same result was found with the endotracheal tube, the best technic was the Digest-EUR® treatment with 3.05 × 107 CFU/ml (SD: 2.35 × 107 CFU/ml), the Digest-EUR® dislodged 3.05 × 107 CFU/ml (2.35 × 107 CFU/ml) and the combination of Digest-EUR® and US was inferior with 3.59 × 106 CFU/ml (SD: 1.72 × 106 CFU/ml).

Combination treatments always started with 15 min of dithiothreitol. Thiol agents separate disulfide bridges from proteins and thus release biofilm fragments. Then, US treatment was carried out for one minute. The multiple microscopic shocks caused by the US after an initial chemical treatment like the Digest-EUR® might cause new 3D conformation of the biofilm fragments limiting the number of quantifiable viable bacteria which possibly aggregate.

These results demonstrate the importance of the physical treatment applied to detach the biofilm to precisely analyze the number of bacteria present in the biofilm. Depending on the materials used, the biofilm dislodging technique must be adapted. Ultrasound has shown its superiority with silicone, PIV, and picc-line implants. However, the best detachment technique on endotracheal tubes is a chemical treatment: the dithiothreitol Digest-EUR®. Silicone, PIV, and piccline materials are flexible, unlike the tracheal tube. The US seems to be more efficient on flexible materials. Precedent studies reported ultrasonic bath treatment was superior to vortexing and direct ultrasonic disruption on vascular prosthetic grafts [60].

The results of this study can motivate new research on other bacterial biofilms including polymicrobial biofilm. It remains unknown whether the ability of sonication or chemical methods for biofilm dislodgement would differ in more mature biofilms, for example, in the clinical setting when dealing with chronic implant-associated infections occurring after long period.

## Conclusion

This study demonstrates that sonication is superior to the chemical method for dislodgement of bacterial biofilms of S. epidermidis on flexible materials as silicone, PIV, and picc-line. The result for a rigid endotracheal tube was different with superiority for the chemical treatment dithiothreitol: Digest-EUR®. These results demonstrate the importance of the physical treatment applied to detach the biofilm to precisely analyze the number of bacteria present in the biofilm. Depending on the materials used, the biofilm dislodging technique must be adapted. Nevertheless the literature provides many discordant results probably due to variation in the sample handling. Scientists should compare themselves different methods before designing a protocol of biofilm study on a given material.

## Materials and methods

### Medical implant

**Fig. 12:**
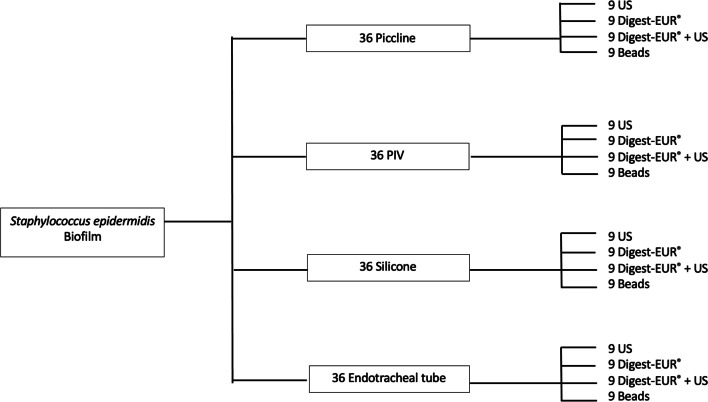
Experimental Flow chart

Four different types of medical implants were tested. For the silicone implants, patches of 1cm^2^ smooth silicone implant Allergan® were used for the experiment. Picc-lines were made with polytetrafluoroethylene 1 cm long tube-shaped implants (Terumo® by Smith medical). Peripheral venous catheter was Ocrilon® polyurethane tube-shaped implants 1 cm long (IV protection by Smith medical). Sample of 1 cm of length of endotracheal tube Rüsch®, Teleflex 7,5 mm of diameter made with polyvinylchloride were used for the experiment. The experiment was repeated 3 times in the same conditions (Fig. 12) with 36 implants of each material tested.

### Biofilm formation

A clinical isolate of *Staphylococcus epidermidis* previously isolated from a breast implant capsular contracture on a patient in the university hospital of Dijon was selected for its ability to produce biofilm on the implants. It is a laboratory collection strain for which only the origin of isolation is known but it is dissociated from the patient's name (anonymization). This anonymization procedure is approved by the university hospital of Dijon and used routinely. It was grown on tryptic soy broth at 37 °C. Overnight cultures were placed in exponential phase and then diluted at 0,5 of OD500 nm, then tenfold diluted. This corresponds to 5 X 10^6^ CFU/ml.

Two analyses were carried out, one young biofilm during 14 h and one mature biofilm during 5 days. For the 5 days biofilm formation, we add 10 cc of tryptic soy broth every day.

**Fig. 13:**
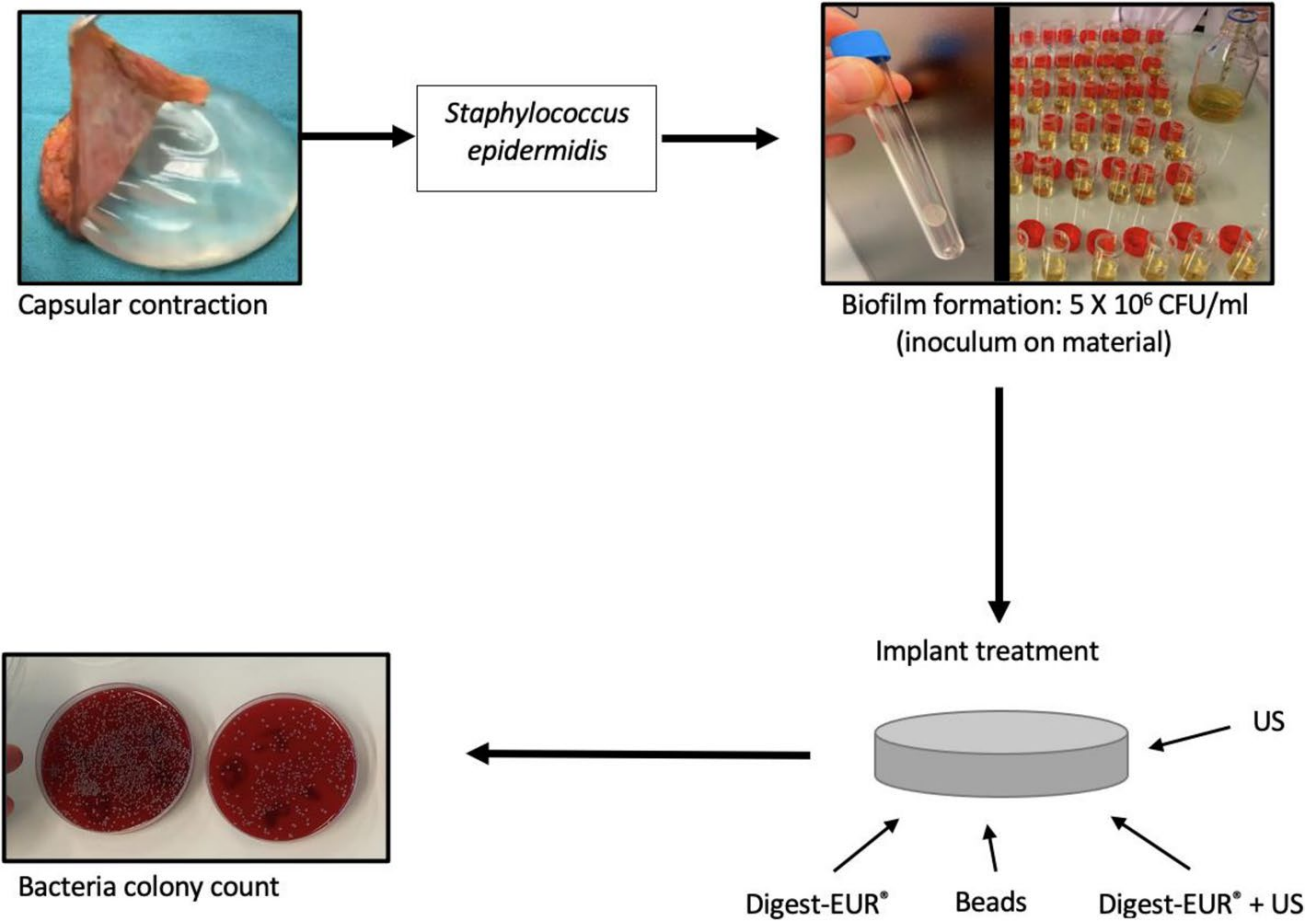
Experimental plan

The implants were incubated at 37 °C (Fig. 13).

### Chemicals and reagents

Chemicals and reagents used for bacteria growth and biofilm detection were purchased from Sigma- Aldrich (Switzerland) if not mentioned elsewhere.

### Biofilm removal treatment

Different technics were tested and compared to detach the biofilm and quantify the bacteria.

All the Implants were incubated individually for 14 h at 37 °C in a conical tube. Then, the implants were moved in a new test tube gently washed in 10 ml of PBS. After the washing, the implants were placed in their experimental tube.

Each experimental tube contains 3 ml of sterile distilled water except for the Digest-EUR® tube with only the 3 ml of Digest-EUR® solution. The resulting supernatant was used for CFU counting. Microscopic screening for residual bacteria on the surfaces was conducted. Each material was tested with each biofilm dislodging technique. Nine implants were tested for each of the 4 conditions.

Serial dilution, plating and colony enumeration assumes that the biofilm is truly dispersed into individual cells. Flow cytometry (Sysmex®) [61] was used to characterize the size distribution of material to confirm the single cell dispersal.

- ***US:*** External ultra-sonification was done. The test tube with the distilled water and the implants were placed into the Branson® ultrasonic bath for 1 min at room temperature (40 kHz frequency) and then vortexed for 30 s.
- ***Beads:*** The implants were placed in a sterile tube with 3 ml of distilled water and 1 mm-diameter stainless steel beads before agitation bead mill (6000 rpm) with the Ultra Turrax® Tube Drive disposal.
- ***Mucolytic:*** Digest-EUR® is a mucolytic composed of dithiothreitol for rapid digestion and mucus fluidification. Thiol agents separate disulfide bridges from proteins releasing biofilm fragments. The Digest-EUR® was ten-fold diluted and 3 ml were introduced with the implant at 37 °C for 15 min to detach the biofilm.
- ***Combination of US and mucolytic:*** Combination treatments always started with 15 min immersion in Digest-EUR®. Then, US treatment was carried out for one minute.

### Microscopic analyses

Microscopic analyses were performed to ensure the presence of biofilm on the surface and to check the efficiency of biofilm detachment.

- ***Optical microscopy:*** After incubation and treatment as described above, each implant was fixed with ethanol 90% for 5 min and then rinsed into sterile water. A crystal violet coloration was done during 5 min then rinsed 3 times 5 min into water. The specimen was dried before optical microscopy analysis.
- ***Confocal scanning laser microscopy:*** The area of focus is scanned across the sample to produce high-resolution 2-D “slices” at various heights that are assembled to create a final 3D image [62]. After biofilm formation and detachment as described above, each implant was fixed with 5% glutaraldehyde and gently washed three times 5 min with phosphate-buffered saline (PBS) [63] and then samples were oven drying at 37 degrees. The equipment used was a Caliber I.D. StableView™ 30x magnification 0.9 NA with water immersion and 750 µm x 750 µm scope without stain.

- ***Scanning Electron Microscopy:*** After incubation and treatment as described above, each implant was fixed with 5% glutaraldehyde and gently washed three times 5 min with phosphate-buffered saline (PBS) and then samples were oven drying at 37 degrees. Observations were performed at 15 kV with a scanning electron microscope (model S3500N; Hitachi®, Tokyo, Japan). Five fields of view at magnifications from X500 to X600 were chosen randomly from the optical surface of each sample. Each experiment was conducted in triplicate.

### Bacterial quantification of the biofilm

- Bacterial counting of the removed biofilm: the supernatants were serially diluted and plated on tryptic soy agar sheep blood plates. Plates were incubated for 24 h, and the colonies were manually counted to determine the amount of *Staphylococcus epidermidis* (in CFU/ml).
- Indirect quantification with crystal violet staining: biofilm formation can be indirectly assessed by staining with 1% crystal violet and measuring crystal violet absorbance with an optical density at 595 nm, using destaining solution [64].

### Statistical analysis

Data were documented and evaluated with GraphPad Prism 8 software (GraphPad Software, Inc., La Jolla, USA). Three independent experiments were performed for each biofilm removal method. Bacterial counts were recovered from each implant. Quantitative data were presented as mean ± standard deviation (SD). A one-way ANOVA followed by multiple comparisons was done to compare the different detachment methods. Statistical analysis was performed with a significance level of *p* ≤ 0.05.

## Supplementary Information

**Additional file 1: Figure A.** *Staphylococcus epidermidis* aspect on confocal microscopy.**Figure B.** Silicone patch showed natural absorption of crystal violet. **Figure C.**SEM (200 µm) on the left and a SEM (50 µm) picture on the right shows the formation and removal of biofilms (white spots on images) on silicone for *Staphylococcus epidermidis *with beads*. *We can observe the presence of bacteria inside the EPS 3D formation on the right picture. **Table A.** Results of the different treatments in a 14 hours *Staphylococcus epidermidis* biofilm removal on piccline, PIV, silicone and endotracheal tube. **Table B.** Results of the different treatments in a 5 days *Staphylococcus epidermidis* biofilm removal on piccline, PIV, silicone and endotracheal tube.

## Data Availability

The datasets used and/or analysed during the current study are available from the corresponding author on reasonable request.

## Abbreviations

BAI: Biomaterial-associated infection
BIA-ALCL: Breast implants and anaplastic large cell lymphoma
CC: Capsular contracture
CFU: Colony forming units
CLSM: Confocal laser scanning microscopy
DTT: Dithiothreitol
EPS: Extracellular polymeric substances
FDA: US Food and Drug administration
NAC: N-acetyl cysteine
OM: Optical microscopy
PBS: Phosphate-buffered saline
PICC-line: Peripherally inserted central venous catheter
PIV: Peripheral intravenous catheters
PMMA: Poly-méthyl-méthacrylate
SEM: Scanning electron microscopy
US: Ultra-sound
UV: Ultra-violet
