# Supplementary material for: What is the best technic to dislodge Staphylococcus epidermidis biofilm on medical implants?
Source: BMC Microbiol. 2022 Aug 6;22:192. doi: 10.1186/s12866-022-02606-x (PMC9356421; doi:10.1186/s12866-022-02606-x)
Supplement: Supplementary file 1 — Additional file 1: Figure A. Staphylococcus epidermidis aspect on confocal microscopy.Figure B. Silicone patch showed natural absorption of crystal violet. Figure C.SEM (200 µm) on the left and a SEM (50 µm) picture on the right shows the formation and removal of biofilms (white spots on images) on silicone for Staphylococcus epidermidis with beads. We can observe the presence of bacteria inside the EPS 3D formation on the right picture. Table A. Results of the different treatments in a 14 hours Staphylococcus epidermidis biofilm removal on piccline, PIV, silicone and endotracheal tube. Table B. Results of the different treatments in a 5 days Staphylococcus epidermidis biofilm removal on piccline, PIV, silicone and endotracheal tube. [file 12866_2022_2606_MOESM1_ESM.docx]

**Supplemental Material**


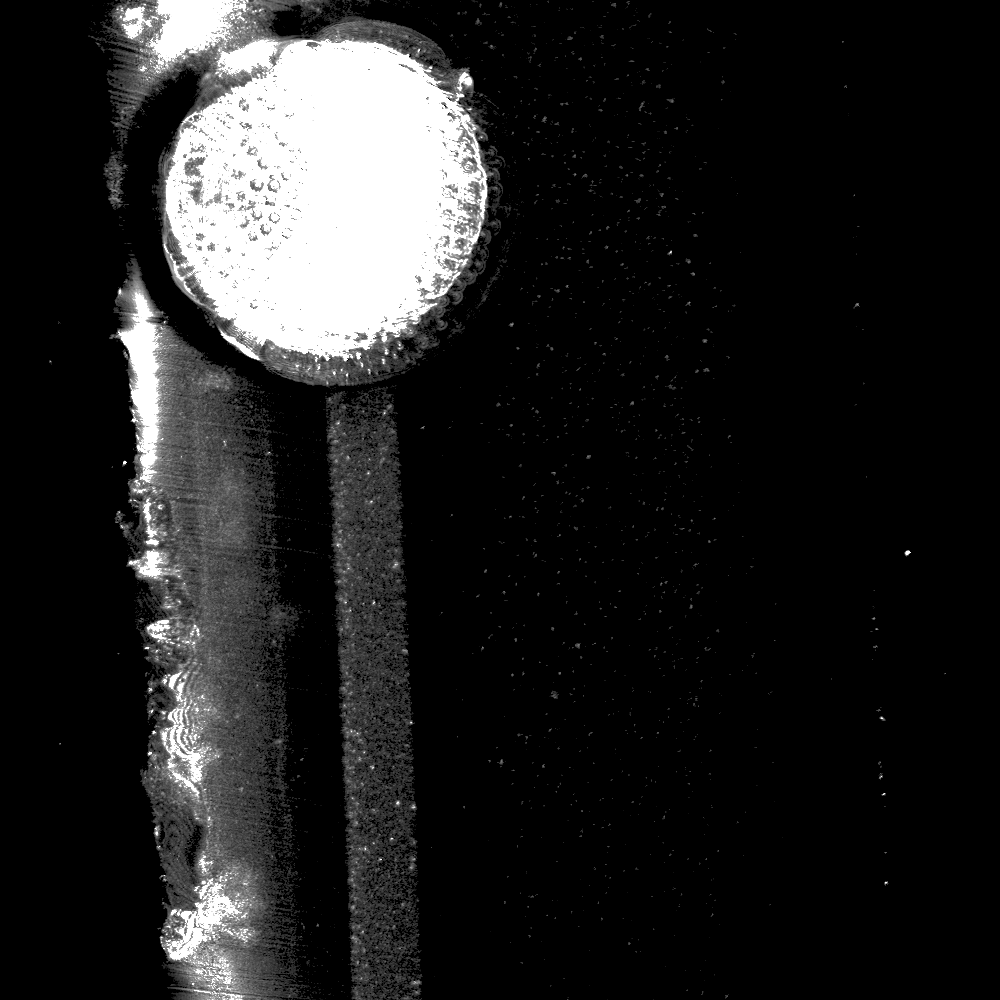


Interior side of the silicone implant

External side of the silicone implant

*Staphylococcus epidermidis* biofilm

Figure A: *Staphylococcus epidermidis* aspect on confocal microscopy


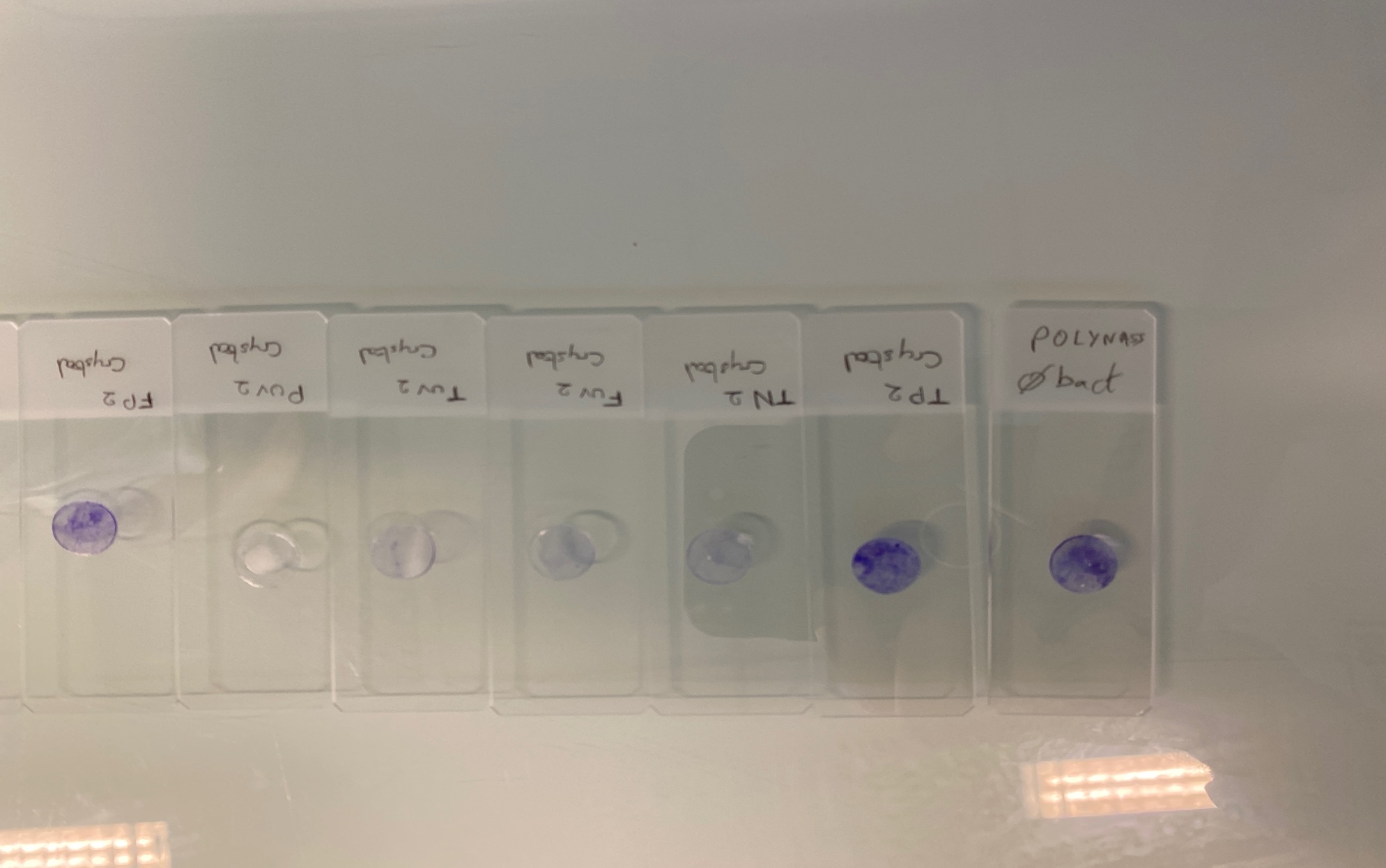


Figure B: Silicone patch showed natural absorption of crystal violet


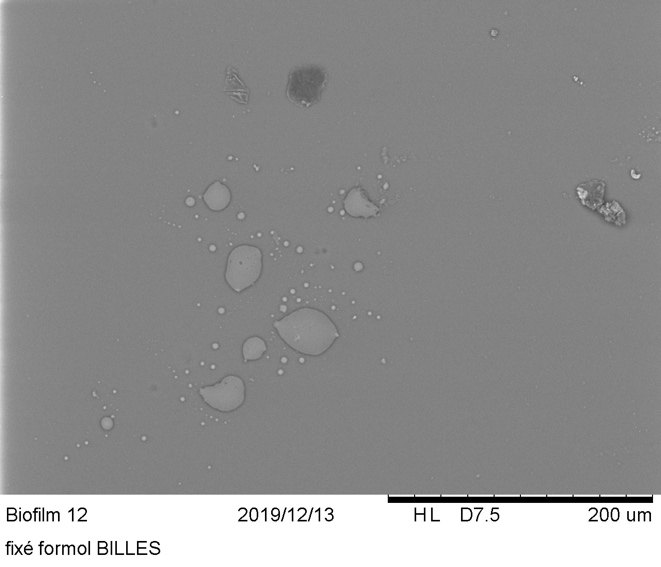

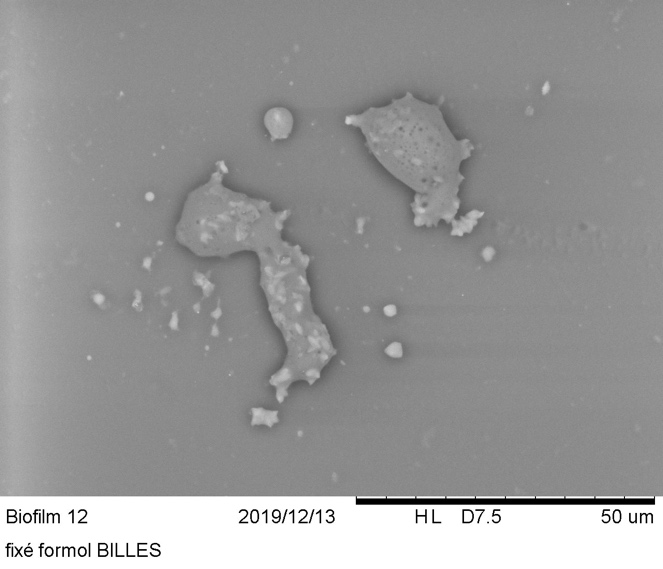


Figure C: SEM (200 µm) on the left and a SEM (50 µm) picture on the right shows the formation and removal of biofilms (white spots on images) on silicone for *Staphylococcus epidermidis* with beads*.* We can observe the presence of bacteria inside the EPS 3D formation on the right picture.

TABLE

| Procedure | Mean (CFU/ml) | SD (CFU/ml) |
| --- | --- | --- |
| **US** | **1.75 x 10^7^** | **7.07 x 10^6^** |
| Digester + US | 4.75 x 10^6^ | 1.04 x 10^6^ |
| Beads | 1.06 x 10^6^ | 1.14 x 10^6^ |
| Digester | 1.9 x 10^6^ | 7.7 x 10^5^ |

Piccline

| Procedure | Mean (CFU/ml) | SD (CFU/ml) |
| --- | --- | --- |
| **US** | **1.45 x 10^7^** | **5.46 x 10^6^** |
| Digester + US | 3.13 x 10^6^ | 1.71 x 10^6^ |
| Beads | 2.55 x 10^6^ | 6.95 x 10^5^ |
| Digester | 5.01 x 10^6^ | 2.17 x 10^6^ |

Peripherical intravenous catheter (PIV)

| Procedure | Mean (CFU/ml) | SD (CFU/ml) |
| --- | --- | --- |
| **US** | **3.59 x 10^7^** | **2.95 x 10^7^** |
| Digester + US | 7 x 10^6^ | 8.25 x 10^6^ |
| Beads | 3.61 x 10^6^ | 3.02 x 10^6^ |
| Digester | 6.36 x 10^6^ | 9.87 x 10^6^ |

Silicone

| Procedure | Mean (CFU/ml) | SD (CFU/ml) |
| --- | --- | --- |
| US | 1.19 x 10^7^ | 6.85 x 10^6^ |
| Digester + US | 3.59 x 10^6^ | 1.72 x 10^6^ |
| Beads | 2.53 x 10^6^ | 1.70 x 10^6^ |
| **Digester** | **3.05 x 10^7^** | **2.35 x 10^7^** |

Endotracheal tube

**Table A:** Results of the different treatments in a 14 hours *Staphylococcus epidermidis* biofilm removal on piccline, PIV, silicone and endotracheal tube.

| Procedure | Mean (CFU/ml) | SD (CFU/ml) |
| --- | --- | --- |
| **US** | **5 x 10^9^** | **2.5 x 10^9^** |
| Digester + US | 1.2 x 10^7^ | 6.3 x 10^6^ |
| Beads | 3.4 x 10^8^ | 1.9 x 10^8^ |
| Digester | 3.03 x 10^6^ | 1.27 x 10^6^ |

Piccline

| Procedure | Mean (CFU/ml) | SD (CFU/ml) |
| --- | --- | --- |
| **US** | **5 x 10^9^** | **1.6 x 10^9^** |
| Digester + US | 1.15 x 10^7^ | 5.41 x 10^6^ |
| Beads | 3.53 x 10^8^ | 1.30 x 10^8^ |
| Digester | 3.02 x 10^7^ | 6.40 x 10^6^ |

Peripherical intravenous catheter (PIV)

| Procedure | Mean (CFU/ml) | SD (CFU/ml) |
| --- | --- | --- |
| **US** | **8.63 x 10^9^** | **1.22 x 10^9^** |
| Digester + US | 3.01 x 10^8^ | 1.33 x 10^8^ |
| Beads | 2.74 x 10^8^ | 1.20 x 10^8^ |
| Digester | 4.52 x 10^8^ | 1.70 x 10^8^ |

Silicone

| Procedure | Mean (CFU/ml) | SD (CFU/ml) |
| --- | --- | --- |
| US | 5.34 x 10^8^ | 1.06 x 10^8^ |
| Digester + US | 7.41 x 10^8^ | 1.22 x 10^8^ |
| Beads | 3.18 x 10^8^ | 9.73 x 10^7^ |
| **Digester** | **6.91 x 10^9^** | **1.43 x 10^9^** |

Endotracheal tube

**Table B :** Results of the different treatments in a 5 days *Staphylococcus epidermidis* biofilm removal on piccline, PIV, silicone and endotracheal tube.
